# Different inflammation responses modulate Müller glia proliferation in the acute or chronically damaged zebrafish retina

**DOI:** 10.3389/fcell.2022.892271

**Published:** 2022-08-31

**Authors:** Maria Iribarne, David R. Hyde

**Affiliations:** ^1^ Department of Biological Sciences, University of Notre Dame, Notre Dame, IN, United States; ^2^ Center for Zebrafish Research, University of Notre Dame, Notre Dame, IN, United States; ^3^ Center for Stem Cells and Regenerative Medicine, University of Notre Dame, Notre Dame, IN, United States

**Keywords:** regeneration, inflammation, microglia, cytokines, Müller glia, retina, genetic mutant, zebrafish

## Abstract

Unlike mammals, zebrafish regenerate in response to retinal damage. Because microglia are activated by retinal damage, we investigated their role during regeneration following either acute or chronic damage. At three weeks post-fertilization (wpf), both wild-type fish exhibiting NMDA-induced acute ganglion and amacrine cell death and *gold rush (gosh)* mutant fish possessing chronic cone photoreceptor degeneration displayed reactive microglia/macrophages and Müller glia proliferation. Dexamethasone-treated retinas, to inhibit the immune response, lacked reactive microglia/macrophages and possessed fewer PCNA-positive cells, while LPS treatment increased microglia/macrophages and PCNA-labeled cells. NMDA-injured retinas upregulated expression of *il-1β* and *tnfα* pro-inflammatory cytokine genes, followed by increased expression of *il-10* and *arg1* anti-inflammatory/remodeling cytokine genes. A transient early TNFα pro-inflammatory microglia/macrophage population was visualized in NMDA-damaged retinas. In contrast, *gosh* mutant retinas exhibited a slight increase of pro-inflammatory cytokine gene expression concurrently with a greater increased anti-inflammatory/remodeling cytokine gene expression. Few TNFα pro-inflammatory microglia/macrophages were observed in the *gosh* retina. Understanding why acute and chronic damage results in different inflammation profiles and their effects on regulating zebrafish retinal regeneration would provide important clues toward improving therapeutic strategies for repairing injured mammalian tissues.

## Introduction

Most vertebrates, including humans, cannot to regenerate retinal neurons that are lost due to traumatic injury or degenerative disease. In contrast, lower-order vertebrates, such as zebrafish, possess an extraordinary regenerative ability, which restores both the lost neurons and normal function to the damaged retina (Iribarne, 2019; Hoang et al., 2020; Hammer et al., 2021). Upon neuronal loss in the zebrafish retina, Müller glia reprogram to a retinal progenitor cell-like state and re-enter the cell cycle to generate neuronal progenitor cells (NPCs). These NPCs continue to proliferate and migrate to the site of neuronal damage and primarily differentiate into the neuronal types that were lost (Yurco and Cameron, 2005; Fausett and Goldman, 2006; Bernardos et al., 2007; Fimbel et al., 2007). Recent studies in the mouse retina stimulated a small Müller glia proliferative response and generation of new neurons after either inducing or repressing expression of specific transcription factors, which were identified in the zebrafish retinal regenerative response (Jorstad et al., 2017; Elsaeidi et al., 2018; Yao et al., 2018; Hoang et al., 2020). Thus, understanding mechanisms by which zebrafish can regenerate lost neurons may provide strategies for stimulating mammalian retina regeneration.

Recent evidence suggests that the innate immune system can modulate the regenerative response following zebrafish neuronal damage. Acute and transient inflammation is necessary to induce a regenerative response in the adult zebrafish telencephalon (Kyritsis et al., 2012). Co-depleting or inhibiting microglia functions prior to rod photoreceptor ablation in zebrafish larvae blocked the Müller glia regenerative response (White et al., 2017). Similarly, pharmacological treatment with either dexamethasone or PLX3397 prior to various retinal injuries reduced the number of proliferating Müller glia and neuronal progenitor cells in adult zebrafish (Conedera et al., 2019; Silva et al., 2020; Zhang et al., 2020). Although the involvement of inflammation during retinal regeneration has been reported, its molecular mechanism in modulating Müller glia proliferation remains elusive.

Inflammation is a dynamic process that involves recruiting inflammatory cells and secretion of pro-inflammatory cytokines and molecular mediators. The resolution of inflammation is critical to avoid tissue damage (Bollaerts et al., 2017; Bosak, Murata et al., 2018; Iribarne, 2021). M1-like macrophages are pro-inflammatory cells associated with the first phase of inflammation and typically express IL-1β and TNFα; while M2-like macrophages are involved in the resolution of inflammation and tissue remodeling response and express IL-10 and TGF-β1 (Nguyen-Chi et al., 2015). The differential expression of cytokines and chemokines, as well as the expression of receptors, defines the polarization state of macrophages. Several zebrafish studies suggest that activation and duration of pro-inflammatory signals and the subsequent resolution are critical in creating an instructive microenvironment for tissue regeneration. For instance, a transient inflammatory response mediated by IL-1β is required for proper regeneration of the zebrafish fin fold, where macrophages are responsible for attenuating IL-1β expression (Hasegawa et al., 2017). Similarly, an interplay between IL-1β and TNFα from neutrophils and macrophages is necessary for regeneration of the injured spinal cord in zebrafish larvae (Tsarouchas et al., 2018). However, it remains unknown what cytokine profiles are expressed during the regeneration process and whether microglia switch from pro-inflammatory to resolution state in the damaged retina.

Most previous retinal regeneration studies employed acute damage in the adult retina, such as intense light exposure (Vihtelic and Hyde, 2000; Bernardos et al., 2007), retinal puncture (Fausett and Goldman, 2006), chemical ablation (Fimbel et al., 2007; Powell et al.,2016), or ectopic expression of a toxic transgene, such as nitroreductase (Montgomery et al., 2010; Hagerman et al., 2016). Acute damage leads to rapid retinal cell loss that resembles traumatic injury in human patients. Alternatively, retinal regeneration studies using chronic damage models in zebrafish are limited (Morris et al., 2008; Nishiwaki et al., 2008; Sherpa et al., 2011; Iribarne et al., 2017; Iribarne et al., 2019; Song et al., 2020; Turkalj et al., 2021). These chronic degeneration models are often genetic-based and display a slower loss of retinal neurons like human genetic diseases. They can begin to show signs of retinal damage either in development or in adulthood. Acute retinal damage in zebrafish usually induces a Müller glia-dependent regenerative response, while chronic retinal damage is more variable. Chronic retinal damage in some injury models exhibit rod precursor cell proliferation and not Müller glia-dependent regeneration (Morris et al., 2008; Song et al., 2020; Turkalj et al., 2021), while some mutants possess Müller glia-dependent regeneration (Morris et al., 2008; Nishiwaki et al., 2008; Iribarne et al., 2019). While several acute damage studies have focused on the role of inflammation during the regeneration process, similar studies using chronic retinal models have not been reported. Therefore, we examined the regenerative response following acute and chronic retinal damage with a focus on the role of inflammation. Understanding how inflammation regulates regeneration in either acute or chronic retinal damage in zebrafish would provide important insights to improve the therapeutic strategies for repairing injured mammalian tissues that do not have an inherent regenerative capacity.

## Materials and methods

### Fish

Zebrafish (*Danio rerio*) were maintained in the Center for Zebrafish Research at the University of Notre Dame Freimann Life Science Center using standard procedures (Westerfield, 1993). All the zebrafish larvae (regardless of genetic background) were fed rotifers from 4 dpf to 12 dpf to overcome the proliferation delay that we previously observed with the *gosh* mutant at 3 wpf. AB wild-type fish were used as a wild-type strain. The *gold rush* (*gosh)* mutant was originally isolated in a screen for zebrafish visual mutants using a chemical mutagen, N-ethyl-N-nitrosourea (ENU) (Muto et al., 2005). Zebrafish transgenic lines *Tg(mpeg1:eGFP)* or *Tg(mfap4:tdTomato-CAAX)* were used to monitor microglia behavior (Ellett et al., 2011; Walton et al., 2015), which were generated by the Masai lab using constructs from the Lieschke and Tobin labs (Ranawat and Masai, 2021). *Tg(gfap:eGFP)* ^*nt11*^ was used to visualize Müller glia (Kassen et al., 2007), *TgBAC(tnfα:GFP)* ^*pd1028*^ (Marjoram et al., 2015) was used to identify *tnfα-*expressing pro-inflammatory macrophages, and *Tg(mpeg1:NTR-eYFP)* ^*w202*^ was used to ablate macrophages (Petrie et al., 2014). Because the adaptive immune system develops around 4–6 wpf in zebrafish (Yoder et al., 2002), we primarily used 3 wpf juvenile larvae to study the innate immune system response in the two retinal injury models, except when indicated. Fish were euthanized by an anesthetic overdose of 0.2% 2-phenoxyethanol, and eyes were enucleated for further processing. All experimental protocols were approved by the animal use committee at the University of Notre Dame and followed the National Institutes of Health guide for the care and use of Laboratory animals (NIH Publications No. 8023, revised 1978).

### Optokinetic response (OKR) behavior test

*gosh* visual mutants used in this study were obtained from incrossing *gosh* carriers and screened at 5–7 days post-fertilization (dpf) using the OKR test to identify them from wild-type and heterozygous siblings (Iribarne et al., 2017). In a petri dish containing methylcellulose, 10 wells were filled with aquarium water in which the fish were raised to minimize stress to the fish. Individual larvae were partially immobilized in single wells and examined under a stereoscopic microscope. To evaluate visual acuity, a drum with black and white vertical stripes (at 18 separation) was placed around the petri dish and spun at 10–20 rpm. Larval eye movement was observed under the stereoscopic microscope to identify cone blind fish.

### NMDA-induced acute damage

Death of ganglion and amacrine retinal neurons was induced by a single intravitreal injection of 0.5–1 nL of freshly prepared 100 mM N-Methyl-D-aspartic acid (NMDA, M3262, Sigma) in water. Briefly, fish were anesthetized in 0.1% 2-phenoxyethanol, and under microscopic visualization, NMDA was delivered using a FemtoJet express microinjector (Eppendorf). Control injections were sterile water. Retinas that were coinjected with LPS and NMDA were injected at 0 h with LPS, followed by NMDA injection at 3 h. Retinas were then collected at 72 h following LPS injection.

### Drug treatment

Dexamethasone (Dex, D1756, Sigma-Aldrich) was diluted in 100% methanol to generate a 5 mM stock concentration. Fish were placed in tanks containing 5 μM Dex in aquarium water, with the solution changed daily. Control fish were maintained in tanks containiong 0.025% methanol. *Escherichia coli* lipopolysaccharides O55:B5 (LPS, L2880, Sigma-Aldrich) were dissolved in PBS to a concentration of 1 mg/ml. One single intravitreal injection of 0.5–1 nL was carried out using a FemtoJet express microinjector (Eppendorf). Control injections were 1x PBS.

### Macrophage ablation

To ablate macrophages, the transgenic line *Tg(mpeg1:NTR-eYFP)* ^*w202*^ was used (Petrie et al., 2014). 5 mM Metronidazole (MTZ, M3764, Sigma-Aldrich) was prepared fresh in aquarium water in a light-tight tank. 3 wpf fish, both control and experimental, were placed in tanks containing MTZ, with the 5 mM solution changed daily. Fish were kept in the tanks with MTZ for 4 or 5 days.

### Histology

3 wpf heads were fixed with 4% paraformaldehyde in 0.1 M phosphate buffer (4% PFA, Sigma-Aldrich), pH 7.3 overnight at 4°C or in 9:1 ethanol:formaldehyde (Fisher Scientific) for 2 h at room temperature on a shaker. Heads were washed either three times in PBS (for 4% PFA fix) or an ethanol gradient followed by PBS (for 9:1 ethanol:formaldehyde fix). Heads were then cryoprotected and rapidly frozen (Masai et al., 2003). Immunolabeling of cryosections (14 μm thickness) was performed as previously described (Masai et al., 2003). For antigen retrieval, cryosections were pretreated with heat (∼90°C, 10 min, in 10 mM citrate buffer, pH 6.0). Mouse anti-PCNA antibody (clone PC10, Sigma P8825; 1:1,000) and mouse anti-4C4 (HPC Cell Cultures, 92092321, 1:50) were used. Anti-GFP (Life Technology, A11122, 1:500) and anti-RFP (Rockland, 600-401-379, 1:200) antibodies were used to amplify the GFP and tdTomato-CAAX signal after antigen retrieval. Nuclear staining was performed using 5 μg/ml DAPI (Invitrogen). Images were acquired using a Nikon A1r confocal laser scanning microscope.

### Quantitative real-time PCR

RNA was isolated from 3 wpf NMDA-treated wild-type fish, *gosh* mutants, and control fish. 12–15 fish heads were dissected and pooled. RNA was extracted using TRIzol reagent (Life Technologies). Total cDNA was synthesized from 1 μg of RNA using qScript cDNA SuperMix (Quanta Biosciences, Gaithersburg, MD). Reactions were assembled using PerfeCta SYBR Green SuperMix (ROX; Quanta Biosciences). Primers used in this study are included in Table 1. Data were acquired using the StepOnePlus Real-Time PCR system (Applied Biosystems, Foster City, CA, United States). Analysis was performed using the Livak 2^−ΔΔC(t)^ method (Johnson et al., 2000; Vong et al., 2003).

**TABLE 1:** Primers used in this study for qPCR experiments.

Gene	Forward 5′ to 3′	Reverse 5′ to 3′	Literature
***mpeg1***	5′-CAT​GTC​GTG​GCT​GGA​ACA​GA-3′	5′-ATG​GTT​ACG​GAC​TTG​AAC​CCG-3′	Mitchell et al., (2019)
***p2ry12***	5′-AGC​GTC​TCC​AAC​AGT​TCA​TCC-3′	5′-GCC​AGA​GCG​TTC​AGG​GAT​AAT​C-3′	Mitchell et al., (2019)
***il-1β***	5′-CGC​TCC​ACA​TCT​CGT​ACT​CAA-3′	5′-AAC​AGC​AGC​TGG​TCG​TAT​CC-3′	Hui et al., (2017)
***tnf-α***	5′-AGG​CAA​TTT​CAC​TTC​CAA​GG-3′	5′-AGG​TCT​TTG​ATT​CAG​AGT​TGT​ATC​C-3′	Hui et al., (2017)
***tnf-β***	5′-CGA​AGA​AGG​TCA​GAA​ACC​CA-3′	5′-GTT​GGA​ATG​CCT​GAT​CCA​CA-3′	Nguyen-Chi et al., (2015)
***il-10***	5′-CTT​TGC​GAC​TGT​GCT​CAG​AG-3′	5′-TGG​TTC​CAA​GTC​ATC​GTT​GGA​C-3′	Hui et al., (2017)
***tgf-β1***	5′-CAA​CCG​CTG​GCT​CTC​ATT​TGA-3′	5′-ACA​GTC​GCA​GTA​TAA​CCT​CAG​CT-3′	Nguyen-Chi et al., (2015)
***arg1***	5′-ACG​GCC​AGC​CGA​TGT​CTT​AC-3′	5′-TCC​ACG​TCT​CGG​AGT​CCA​AT-3′	Nguyen-Chi et al., (2015)
***ccr2***	5′-TGG​CAA​CGC​AAA​GGC​TTT​CAG​TGA-3′	5′-TCA​GCT​AGG​GCT​AGG​TTG​AAG​AG-3′	Nguyen-Chi et al., (2015)
***18s***	5′-AAT​TGA​CGA​AGG​GCA​CCA​C-3′	5′-CTA​AGA​ACG​GCC​ATG​CAC​CA-3′	Lahne et al., (2015)

### Quantifying cells

We quantified the number of PCNA-labeled cells in the outer nuclear layer (ONL) and the inner nuclear layer (INL), or GFP-expressing cells in the inner retinal layers (including cells in the GCL, IPL, and INL) or all the retina layers of the *Tg(mpeg1:eGFP)* transgenic line, throughout the depth of the z-stack (8–10 μm thickness) in either a 300 μm length of vehicle-injected and NMDA-damaged retinas; or the entire retinal section for control sibling and *gosh* mutants. Cells that were PCNA- and GFP-positive labeled were not quantified (these cells are microglia/macrophages dividing cells) and are not displayed in the PCNA quantification panels. Each experiment was quantified over a total of at least two independent trials, with 3–7 fish per trial, and the average number of labeled cells and SEM were calculated. The statistical test used to analyze the data for each experiment is described in the corresponding figure legend. Graphs were generated using Graphpad Prism9 software and Excel.

## Results

### Acute and chronic retinal damage stimulates microglial activation and Müller glia proliferation

We took advantage of two different neuronal injury paradigms to investigate whether inflammation regulates Müller glia proliferation. We used a NMDA-mediated excitotoxicity model that selectively damages amacrine cells and ganglion cells in the adult zebrafish inner nuclear layer (INL) and ganglion cell layer (GCL), respectively, but spares photoreceptors to generate an acute injury (Powell et al., 2016; Luo et al., 2019). Alternatively, the genetic mutant *gosh*, which exhibits a progressive cone photoreceptor degeneration and regeneration, was used as a chronic retinal damage model (Iribarne et al., 2017; Iribarne et al., 2019). Notably, larval zebrafish possess only an innate immune system, which allows for the study of innate responses in isolation. In contrast, the adaptive immune system develops around 4–6 wpf (Yoder et al., 2002). Thus, we used 3 wpf fish to study the innate immune system response in the two retinal injury models. We used the transgenic lines *Tg(mpeg1:GFP)* and *Tg(mfap4:tdTomato-CAAX)* that express GFP or tdTomato specifically in macrophages, respectively, to monitor the macrophage (re)activation response (Ellett et al., 2011; Walton et al., 2015). Because these transgenic lines cannot distinguish retinal microglia, which are the resident retinal macrophage, from peripheral macrophages, we will refer to these cells as microglia/macrophages.

**FIGURE 1:**
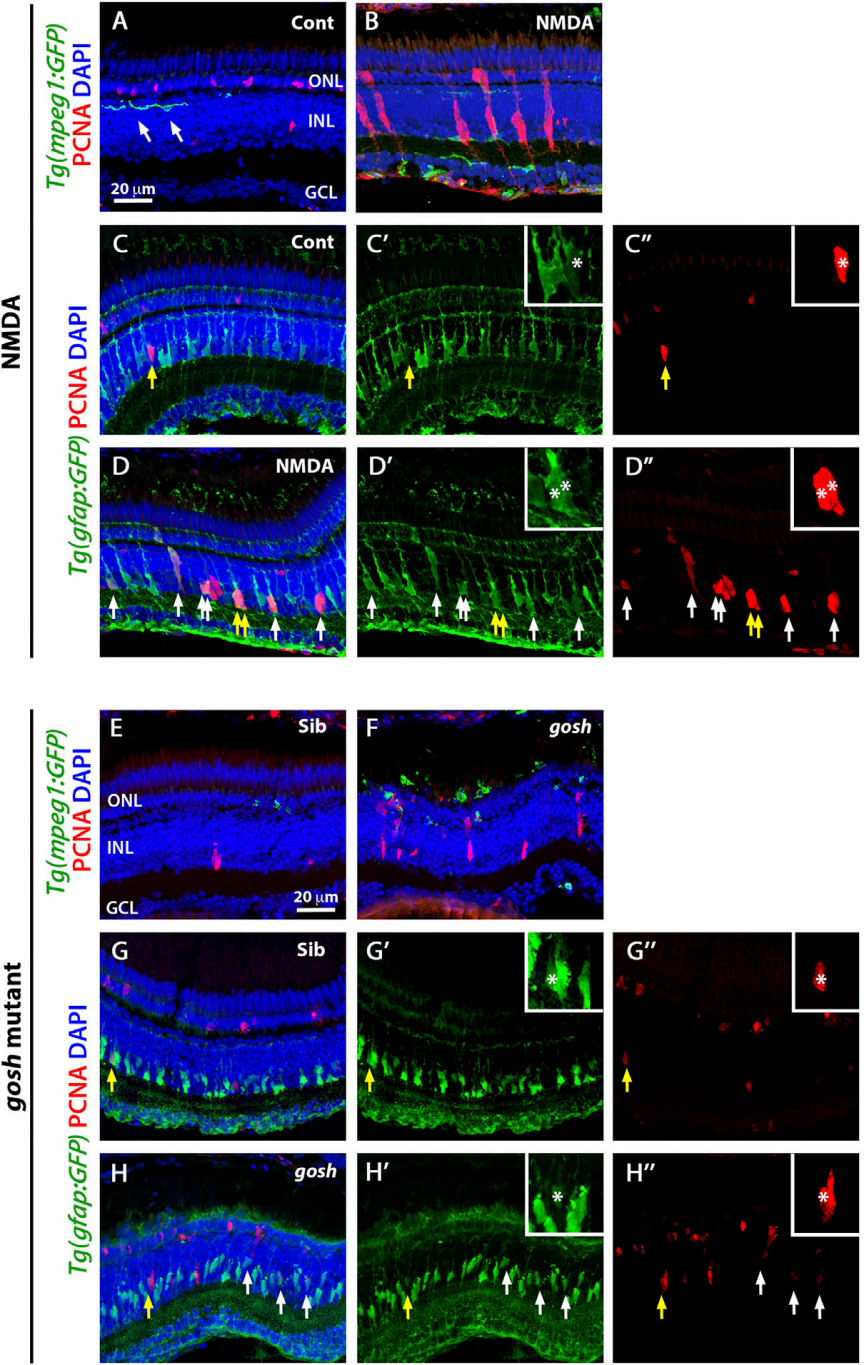
Acute and chronic damaged retinas induce microglia activation and Müller glia proliferation. At 3 wpf, undamaged *Tg(mpeg1:eGFP)* fish retinas display few thin and ramified microglia/macrophages (arrows) and PCNA-positive cells in the ONL and INL, corresponding to rod precursors and Müller glia/NPCs, respectively **(A)**. In contrast, 72 h after NMDA injection of *Tg(mpeg1:eGFP)* fish, microglia/macrophages increase in numbers and ameboid-shaped, migrate to the inner retina, and express PCNA strongly in the INL **(B)**. Forty-eight hours after injection, control and NMDA-injured *Tg(gfap:eGFP*) retinas exhibit eGFP expression in Müller glia cell bodies and apical-basal extended processes **(C**–**D”)**. Control retinas possess relatively few PCNA-positive Müller glia resulting from persistent neurogenesis (arrow, **C**–**C”**), while NMDA-injured retinas display increased numbers of proliferating Müller glia (arrows, **D**–**D”**). Similar to control retinas, 3 wpf control *gosh* sibling retinas possess some ramified microglia/macrophages and few PCNA-positive cells **(E)**, while *gosh* mutant retinas have activated microglia/macrophages in the ONL **(F)**. The control *gosh* sibling retinas display proliferating Müller glia and ONL cells **(G**–**G”)**, as did the *gosh* mutant retinas (**H**–**H”**, arrows). All sections are counterstained with DAPI to visualize the three nuclear layers. Arrows in **(C**–**D”)** and **(G**–**H”)** identify PCNA and eGFP colabeled cells; yellow arrows: identify cells shown in the insets; asterisks in the insets: PCNA and eGFP colabeled cells; ONL, outer nuclear layer; INL, inner nuclear layer; GCL, ganglion cell layer.

At 3 wpf, control retinas showed GFP-positive microglia/macrophages in different retinal layers and displayed a ramified morphology with long processes (Figure 1A, arrows). At 72 h following intravitreal NMDA injection, there were increased numbers of GFP-expressing cells, primarily in the injured inner retina, which exhibited an ameboid cell shape (Figure 1B). To distinguish between microglia or infiltrated macrophages, we stained with the 4C4 monoclonal antibody that specifically labels microglia, but not peripheral macrophages [Supplementary Figure S1, (Caldwell et al., 2019)]. Control retinas possessed only 4C4-positive microglia, while the NMDA-damaged retina had both microglia and a few recruited 4C4-negative peripheral macrophages.

We also co-labeled control and NMDA-damaged retinas with anti-PCNA antibody, a cell proliferation marker. In control retinas, a small number of PCNA-positive cells were observed in the INL and outer nuclear layer (ONL), which likely correspond to Müller glia and rod progenitor cells, respectively (Figure 1A). These proliferating cells are the source of persistent neurogenesis, where Müller glia divide asymmetrically and infrequently to produce rod progenitor cells, which migrate to the ONL and are committed to differentiate into rod photoreceptors (Nagashima et al., 2013; Lahne et al., 2015). Upon NMDA injection, PCNA-positive cell numbers increased in the INL and ONL (Figure 1B). Clusters of PCNA-positive cells, which are formed by Müller glia and the Müller glia-derived neuronal progenitor cells (NPCs) arranged in a vertical column, could often be observed. To corroborate that these PCNA-positive INL cells correspond to Müller glia, we used the *Tg(gfap:GFP)* line to visualize Müller glia and stained for PCNA at 48 h post-injury (hpi) (Figures 1C–D″). Control retinas exhibited relatively few PCNA-positive Müller glia (yellow arrow), which correspond to persistent neurogenesis, while NMDA-injured retinas possessed several PCNA-positive Müller glia that represent a regenerative response (white arrows).

Previously, we showed that the *gosh* mutant underwent photoreceptor degeneration (Iribarne et al., 2017). *gosh* mutant retinas at 3 wpf displayed a very thin photoreceptor layer, where cones form a discontinuous layer, and the central retina is the worst affected. In our previous characterization of the regeneration process in the *gosh* mutant, we found that Müller glia did not proliferate at 3 wpf, but were proliferating by 5 wpf (Iribarne et al., 2019). In this study, we fed all the zebrafish larvae rotifers from 4 dpf to 12 dpf; under these conditions, the larvae overcame the proliferation delay that we observed previously, with the *gosh* mutant possessing a proliferative response at 3 wpf. Cone-blind *gosh* mutant larvae were isolated from siblings at 5 or 6 dpf using an OKR behavior test. In control sibling retinas, microglia/macrophage were detected mainly in the inner plexiform layer and less frequently in the ONL (Figure 1E). In *gosh* mutant retinas, microglia/macrophage displayed a stronger GFP intensity, a greater number of cells, and were localized primarily in the ONL, where photoreceptors were dying, and in the outer segment region (Figure 1F). The 4C4 antibody staining in the transgenic *Tg(mpeg1:GFP)* fish demonstrated that sibling retinas contained only microglia, while *gosh* mutant retinas possessed mostly microglia and a few peripheral macrophages that infiltrated into the retina at 3 wpf (Supplementary Figure S2). Additionally, 1 wpf, 2 wpf, and 4 wpf *gosh* mutant retina sections showed a strong GFP labeling and ameboid cell shape (Supplementary Figures S3A–F), suggesting that the inflammatory response is not limited to the specific time of 3 wpf.

PCNA immunostaining revealed cells in the INL and ONL that formed small clusters (Figure 1F). The *Tg(gfap:GFP)* line was introduced into the *gosh* background to assess if Müller glia were dividing (Figures 1G–H″. Again, control sibling retinas displayed relatively few PCNA-positive INL and ONL cells. In contrast, *gosh* mutant retinas showed a greater number of PCNA-labeled cells, some of which were in the INL and co-labeled with GFP, indicating that those cells were Müller glia re-entering the cell cycle (Figures 1H–H″, yellow arrow). Taken together, these data revealed that these models of acute and chronic retinal damage induced activation of inflammatory cells and a regenerative response involving Müller glia.

### Dexamethasone or nitroreductase-metronidazole ablation treatment reduces microglial activation and Müller glia proliferation in acute and chronic retinal damage models

**FIGURE 2:**
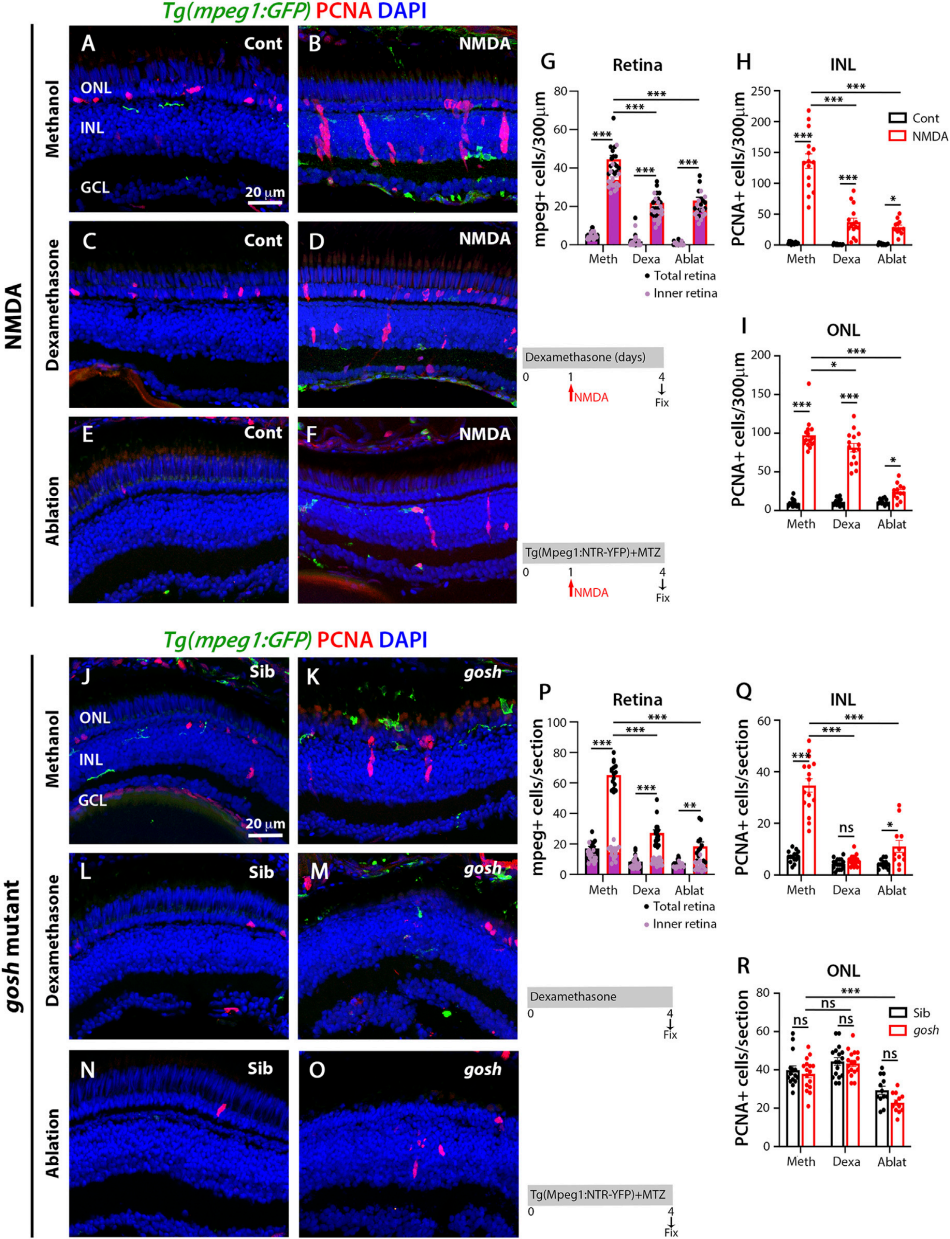
Suppressing microglia affect Müller glia proliferation in NMDA-injured retina and *gosh* mutants. At 72 h after control injection, 3 wpf wild-type *Tg(mpeg1:eGFP)* retinas display few thin and ramified microglia/macrophages through the retina and PCNA-positive cells in the ONL and INL, corresponding to rod precursors and Müller glia/NPCs, respectively **(A)**. At 72 h following NMDA injection, microglia/macrophages increase in numbers in the inner retina, and PCNA expression is strongly stimulated **(B)**. Control and NMDA-damaged retinas were treated with dexamethasone **(C**,**D)** or microglia/macrophages ablated with the *mpeg1:NTR-eYFP* transgene and metronidazole **(E**,**F)**. Compared to methanol-treated control retinas, NMDA-damaged retinas possess significantly fewer microglia/macrophages **(G)** and PCNA-positive INL and ONL cells (**H**,**I**, respectively) following either dexamethasone or ablation treatment. Control *gosh* sibling retinas display some ramified microglia/macrophages and few PCNA-positive cells **(J)**. *gosh* mutant retinas have activated microglia/macrophages in the ONL and PCNA-expressing INL and ONL cells **(K)**. Sibling control and *gosh* mutant retinas were treated with dexamethasone **(L**,**M)** or microglia/macrophages were ablated **(N**,**O)**. Compared to methanol-treated controls, dexamethasone treatment **(M)** or microglia/macrophage ablation **(O)** significantly reduces the number of microglia/macrophages **(P)** and PCNA-positive INL and ONL cells (**Q**,**R**, respectively). Histograms display the number of eGFP-positive microglia/macrophages **(G)** and PCNA-labeled cells **(H**,**I)** in 300 μm of the central region of the NMDA-damaged retinas or in the entire section of *gosh* mutant retinas **(P**–**R)**. Bars and lines indicate mean ± SEM, *n* = 12–17. Black bars: control retinas; red bars: NMDA-injected or *gosh* mutant retinas. Panels **(G**,**P)**: black dots display total number of mpeg:GFP + cells in the retina, and pink dots show total number of mpeg:GFP + cells in the inner retina. One-way ANOVA with Tukey’s multiple comparisons test was applied for all the graphs (ns *p* > 0.05; *p < 0.05; **p < 0.001; ***p < 0.001).

To evaluate whether microglia play a role in the regenerative response following either acute or chronic retinal damage, we used two independent methodologies to suppress microglia/macrophage in the retina: 1) the anti-inflammatory glucocorticoid dexamethasone (Dex) to inhibit microglia/macrophages (White et al., 2017; Silva et al., 2020) and 2) the nitroreductase-metronidazole system to ablate microglia/macrophages (Petrie et al., 2014). The dexamethasone treatment of *Tg(mpeg1:GFP)* transgenic fish started 1 day before either the water or NMDA injection and continued for 4 days. Control retinas possessed few GFP-positive microglia/macrophages, while NMDA-damaged retinas revealed increased numbers of microglia/macrophages mainly located in the inner retina (Figures 2A,B,G; control retinas: 5.43 ± 0.54; NMDA-treated 44.54 ± 1.98; *p* < 0.001). Dex-treated NMDA retinas had a reduced number of microglia/macrophages compared with to water-injected NMDA-damaged group (Figures 2D,G; NMDA + Dex: 21.93 ± 1.45, *p* < 0.001). The control retinas possessed 3.07 ± 0.52 PCNA-positive INL cells and 9.50 ± 1.25 ONL cells (Figures 2A,H,I). Following NMDA injection, the number of PCNA-positive cells increased in both the INL and ONL compared to control retinas (PCNA + NMDA INL: 136.00 ± 11.88, *p* < 0.001, ONL: 97.40 ± 5.39, *p* < 0.001). The Dex-treated retinas also possessed fewer PCNA-positive INL cells, but similar numbers of PCNA-positive ONL cells compared to NMDA-damaged retinas (Figures 2D,H,I; INL: 37.20 ± 6.31, *p* < 0.001, ONL: 81.33 ± 5.43, *p* = 0.06). We ablated macrophages/microglia using the *Tg(mpeg1:NTR-YFP)* line that expresses nitroreductase in macrophages, which leads to cell apoptosis in the presence of the pro-drug metronidazole (MTZ). MTZ treatment started 1 day before either water or NMDA injection and continued for 4 days. NMDA-damaged retinas treated with MTZ displayed fewer macrophages/microglia in the retina than the control NMDA-damaged group (Figures 2F,G; MTZ-treated: 23 ± 1.84, *p* < 0.001, control: 0.58 ± 0.29). The number of PCNA-positive INL and ONL cells was also reduced in these retinas, compared to control NMDA-damaged retinas (Figures 2F,H,I; MTZ-treated INL: 29.08 ± 3.29, *p* < 0.001 and ONL: 24.25 ± 3.05 *p* < 0.001, control INL: 1.25 ± 0.39, ONL: 6.83 ± 0.96).

We used the same strategies to suppress microglia/macrophage in *gosh; Tg(mpeg1:GFP)* transgenic mutant retinas. Control *gosh* sibling retinas possessed 17.07 ± 1.26 GFP-positive microglia/macrophages in all the retinal layers, while *gosh* mutant retinas had 65 ± 2.00 microglia/macrophages in all the retinal layers, but the vast majority located in the outer retina 50.12 ± 1.83 (Figures 2J,K,P). After 4 days of Dex treatment, the *gosh* mutant retinas possessed microglia/macrophages that appeared thinner, ramified, and fewer in number than *gosh* retinas (Figures 2M,P; Dex: 27.2 ± 1.94 GFP-positive cells; *p* < 0.001). Microglia/macrophages also did not accumulate in the ONL or outer segment layer (Figure 2M). The *gosh* mutant retinas displayed a greater number of PCNA-positive INL cells compared to sibling retinas (Figures 2J,K,Q,R; *gosh* mutant INL: 34.69 ± 2.63, *p* < 0.001, ONL: 37.88 ± 2.05, p:0.89; control INL: 7.13 ± 0.56, ONL: 39.8 ± 2.28). The Dex-treated *gosh* mutant retinas showed very few PCNA-labeled INL cells, but maintained a high number in the ONL similar to the Dex-treated control siblings (Figures 2M,Q,R; INL: 5.53 ± 0.50, *p* < 0.001, ONL: 43.35 ± 1.59, *p* = 0.17). The *gosh; Tg(mpeg1:NTR-YFP)* mutant and *gosh* control siblings were treated 4 days with the pro-drug MTZ. Microglia/macrophage ablation significantly reduced the number of microglia/macrophages in the *gosh* mutant retinas (Figures 2O,P: *gosh* mutant ablation: 18.5 ± 2.8, *p* < 0.001). Microglia/macrophages ablated in *gosh* mutant retinas exhibited few PCNA-positive INL and ONL cells (Figures 2O,Q,R; INL: 11.08 ± 2.3, ONL: 22.83 ± 1.48). All these data suggest that either dexamethasone treatment or nitroreductase-MTZ ablation effectively reduced the number of microglia/macrophage cells and the number of proliferating Müller glia following both NMDA-injured acute damage and *gosh* mutant chronic retinal damage.

### LPS increases the number of microglia and proliferating Müller glia in control and injured retinas

**FIGURE 3:**
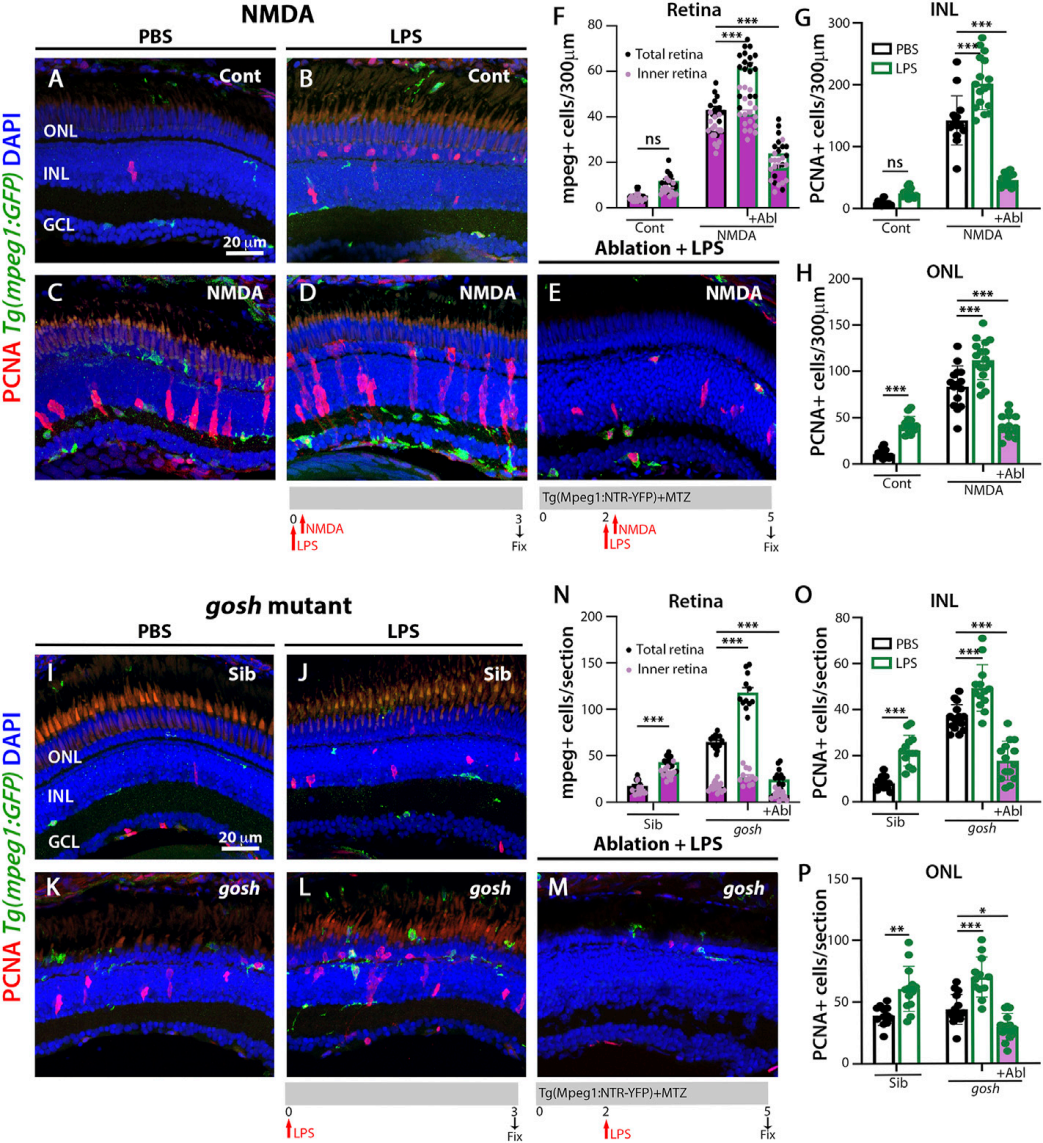
LPS-activated microglia enhance Müller glia proliferation in NMDA-injured retina and *gosh* mutant. Three wpf *Tg(mpeg1:eGFP)* fish with either PBS **(A**–**C)** or LPS **(B**–**D)** and injected 3 h slater with either buffer **(A**,**B)** or NMDA **(C**–**E)** and collected 69 h later for immunostaining. Microglia/macrophages are visualized with the transgenic line. LPS-treated control retinas display a significantly greater number of PCNA-positive ONL cells compared to control retinas **(A**,**B**,**H)**. LPS-treatment of NMDA-damaged retinas significantly increases the numbers of activated microglia/macrophages and proliferating cells compared to PBS-injected NMDA-injured retinas **(C**,**D**,**F**–**H)**. LPS-treatment of NMDA-damaged retinas with ablated microglia/macrophages display significantly fewer microglia/macrophages and PCNA-positive cells **(E**–**H)**. LPS-injected *gosh* mutant eyes significantly increase microglia/macrophages reactivity and PCNA-labeled INL and ONL cells **(K**,**L**,**N**–**P)**. LPS-injected *gosh* mutant fish with ablated microglia/macrophages possess significantly fewer microglia/macrophages and PCNA-positive cells **(M**–**P)**. Histograms display the quantification of the number of eGFP-positive microglia/macrophages **(F)** or PCNA-labeled cells **(G**,**H)** in 300 μm of the central region of the NMDA-damaged retinas or in the entire section of *gosh* mutant retinas **(N**–**P)**. Bars and lines indicate mean ± SEM, *n* = 12–17. Black bars: PBS injection; green bars: LPS injection; green bars with purple fill: LPS and ablated microglia/macrophages. Panels F and N: black dots display total number of mpeg:GFP + cells in the retina, and pink dots show total number of mpeg:GFP + cells in the inner retina. Two-way ANOVA with Tukey’s multiple comparisons test was applied for all the graphs (ns *p* > 0.05; *p < 0.05; **p < 0.01; ***p < 0.001).

To induce inflammation, bacterial lipopolysaccharide (LPS) was injected in *Tg(mpeg1:GFP)* transgenic fish and acute NMDA-damage was induced to assess microglia/macrophage activation and Müller glia proliferation. LPS-treated control retinas showed statistically equivalent numbers of GFP-positive microglia/macrophage compared to PBS-treated control retinas (Figures 3A,B,F; LPS-treated: 11.79 ± 0.99, control: 5.43 ± 0.53, *p* = 0.69). NMDA-induced damage possessed increased numbers of microglia/macrophages after LPS treatment (Figures 3C,D,F; NMDA retinas: 43.07 ± 1.55, NMDA retinas + LPS: 61.53 ± 2.14, *p* < 0.001). In addition, LPS injection increased the number of proliferating Müller glia and NPCs in NMDA-damaged retinas compared to PBS-treated NMDA-damaged retinas (Figures 3B,D,G,H; NMDA + LPS: INL: 201.71 ± 10.129 and ONL: 112.24 ± 5.14, NMDA + PBS INL: 142.53 ± 10.22 and ONL: 83.47 ± 5.80, *p* < 0.001). Because LPS increased the number of PCNA-positive cells in NMDA-injured retinas, we investigated if this phenomenon was microglia/macrophage-dependent. The double transgenic *Tg(mpeg1:NTR-YFP); Tg(mpeg1:GFP)* fish were treated with MTZ to ablate microglia/macrophages. After 2 days of starting MTZ treatment, LPS was injected, and NMDA was injected 3 h later and fish were sacrificed 3 days later. Few microglia/macrophages were observed in MTZ and LPS-treated NMDA-damaged retinas at 72 h after LPS injection (Figures 3E,F; 24 ± 2.48 GFP-positive cells). PCNA-positive cell numbers were strongly reduced in these fish (Figures 3E,G,H; INL: 46.43 ± 2.81, ONL: 41.36 ± 3.24).

We used the same strategy to induce inflammation in *gosh; Tg(mpeg1:GFP)* transgenic mutant retinas *via* injecting LPS. Control *gosh* sibling and *gosh* mutant retinas showed increased numbers of GFP-positive inflammatory cells following LPS injection compared to PBS injection (Figures 3I–L,N; sibling retinas + PBS: 18.08 ± 1.39; sibling retinas + LPS: 43.17 ± 1.89, *p* < 0.001; *gosh* retinas: 64.67 ± 1.88; *gosh* retinas + LPS: 117.92 ± 5.32, *p* < 0.001). PCNA-positive Müller glia and NPCs increased in numbers in sibling and *gosh* mutants upon LPS treatment (Figures 3I–L,O,P; sibling retinas INL: 8.08 ± 0.82 and ONL: 39.08 ± 2.29, sibling retinas + LPS INL: 22.23 ± 1.85, *p* < 0.001 and ONL: 60.62 ± 5.05, *p* < 0.001, *gosh* mutant INL: 36.47 ± 1.48 and ONL: 43.87 ± 3.10, *gosh* mutant + LPS INL: 49.47 ± 2.81, *p* < 0.001 and ONL: 69.92 ± 4.7, *p* < 0.001). The *gosh; Tg(mpeg1:NTR-YFP)* line was treated with MTZ and after 2 days, the fish were injected with LPS (Figure 3M) and fish were sacrificed 3 days later. The nitroreductase-MTZ system allows for efficient microglia/macrophage ablation (Figures 3M,N: *gosh* + LPS + MTZ: 24.29 ± 3.05 GFP-positive cells, *gosh* + LPS: 117.92 ± 5.32, *p* < 0.001). MTZ-treated and LPS-injected *gosh* retinas displayed few PCNA-positive cells (Figures 3M,O,P; INL: 17.86 ± 2.26, ONL: 29.64 ± 2.89). These data suggested that inflammation induces the proliferation of Müller glia in the wild-type retina and potentiates a regenerative response in the damaged retina *via* the action of macrophages/microglia.

### Differential gene expression profile of pro-inflammatory and anti-inflammatory molecules in acute and chronic damage models

**FIGURE 4:**
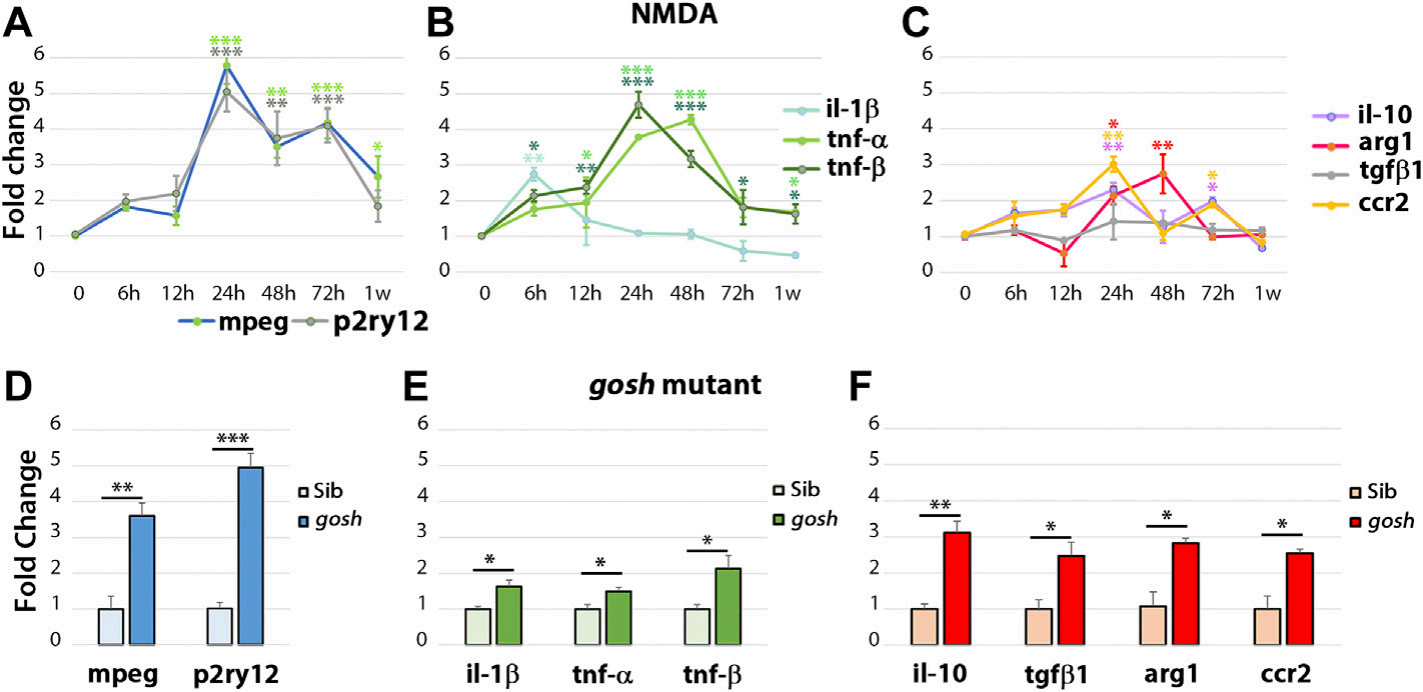
The acute injury model induces pro-inflammatory cytokine gene expressions that switch to anti-inflammatory cytokine expressions, whereas chronic injury shows the simultaneous expression of pro-inflammatory and anti-inflammatory gene expressions. Quantitative real-time PCR was used to determine the fold-change of mRNA expressions of *mpeg1* and *p2ry12* **(A**–**D)**; *il-1β*, *tnfα*, and *tnfβ* **(B**–**E)**; *il-10*, *tgf-β1*, *ccr2*, and *arg1* **(C**,**F)** in NMDA-damaged retinas **(A**–**C)** and *gosh* mutant retinas **(D**–**F)** at 3 wpf. There is significant upregulation of *mpeg1* and *p2ry12* in acute and chronic injured eyes **(A)**. The time course of NMDA-damaged retinas depicts an early *il-1β* peak, followed by stimulation of *tnfα* and *tnfβ* gene expressions, which then decreases within 1 week **(B)**. Anti-inflammatory cytokine gene expressions are induced from 24 hpi, and levels are back to control at 1 week, except for *tgf-β1* that always remains at basal levels **(C)**. *gosh* mutants display a small, but significant upregulation of pro-inflammatory cytokine gene expressions, whereas anti-inflammatory cytokines are upregulated **(D**–**F)**. Graphs represent the mean value of two to three independent experiments ± SEM. For NMDA experiments, a One-way ANOVA with Dunnett’s multiple comparisons test was employed, for *gosh* mutants, an unpaired Student’s t-test was applied. Statistical significance between bars is indicated *p < 0.05, **p < 0.01, ***p < 0.001.

To examine the inflammatory state during the regenerative response in acute and chronic damaged retinas, we assessed relative gene expression levels of several inflammatory cell genes using fish head samples. Expression of *mpeg1* and *p2ry12* genes was used to monitor microglia/macrophage cell activation (Ferrero et al., 2018; Mitchell et al., 2019) and either pro-inflammatory (*il-1β*, *tnfα*, *tnfβ*; (Nguyen-Chi et al., 2015; Tsarouchas et al., 2018; Bollaerts et al., 2019)) or anti-inflammatory/remodeling (*il-10*, *tgf-β1*, *arg1*, *ccr2;* (Nguyen-Chi et al., 2015; Bollaerts et al., 2019)) immune response genes were evaluated. RNAs from NMDA-injured retina at different times (6 h to 1-week post-injection) were used to perform quantitative real-time PCR (qPCR) (Figures 4A–C). NMDA injection induced activation of macrophage/microglia at 24 hpi and persisted through 72 hpi. After 1 wpi, *p2ry12* gene expression returned to control levels while *mpeg1* expression remained significantly higher than control (Figure 4A). In NMDA-injected retinas, expression of pro-inflammatory cytokine *il-1β* gene peaked at 6 hpi (*p* < 0.05) and then returned to control level (Figure 4B). *tnfβ* gene expression increased by 6 hpi and *tnfα* by 12 hpi, their expression peaks persisted through 48 hpi. At 1 week post-injury, *tnfα* and *tnfβ* expression still remained significantly higher than control levels. *il-10* and *ccr2* genes displayed similar expression patterns, with two significantly elevated peaks, one at 24 hpi and a second at 72 hpi. The anti-inflammatory cytokine *arg1* gene was expressed at higher levels than control at 24 and 48 hpi, while *tgf-β1* exhibited similar expression levels to control samples through all the time points evaluated. All the anti-inflammatory genes evaluated in this study were reduced to control levels by 1-week post-injury.

Similar inflammatory markers were assessed in the *gosh* mutant at 3 wpf (Figures 4D–F) and 1, 2, and 4 wpf (Supplementary Figures S3D–L). Inflammatory cell markers were induced in the *gosh* mutant relative to control siblings (Figure 4D; ∼4-fold for *mpeg1*, *p* < 0.01; ∼5-fold for *p2ry12*, *p* < 0.001). Pro-inflammatory cytokine genes were also upregulated, but the significant increase was slight relative to the control siblings (Figure 4E; ∼1.7-fold for *il-1β* and tnfα, *p* < 0.05; ∼2-fold for *tnfβ*, *p* < 0.05). Anti-inflammatory cytokines were all upregulated with a fold increase of approximately 3-fold relative to control siblings (Figure 4F). It is worth noting that *tgf-β1* was upregulated in the chronic damage, but not in the acute NMDA damage, suggesting an expression dependent on the injury paradigm. Hence, in this acute retinal damage model, the immune response is biphasic with an initial pro-inflammatory phase, followed by an anti-inflammatory/remodeling phase. In the chronic *gosh* mutant damage model, both pro-inflammatory and anti-inflammatory cytokines overlap, with pro-inflammatory cytokine genes showing a slight increase and anti-inflammatory genes exhibiting a larger fold increase.

### Microglia express TNFα in acute and chronic retinal damage zebrafish models

**FIGURE 5:**
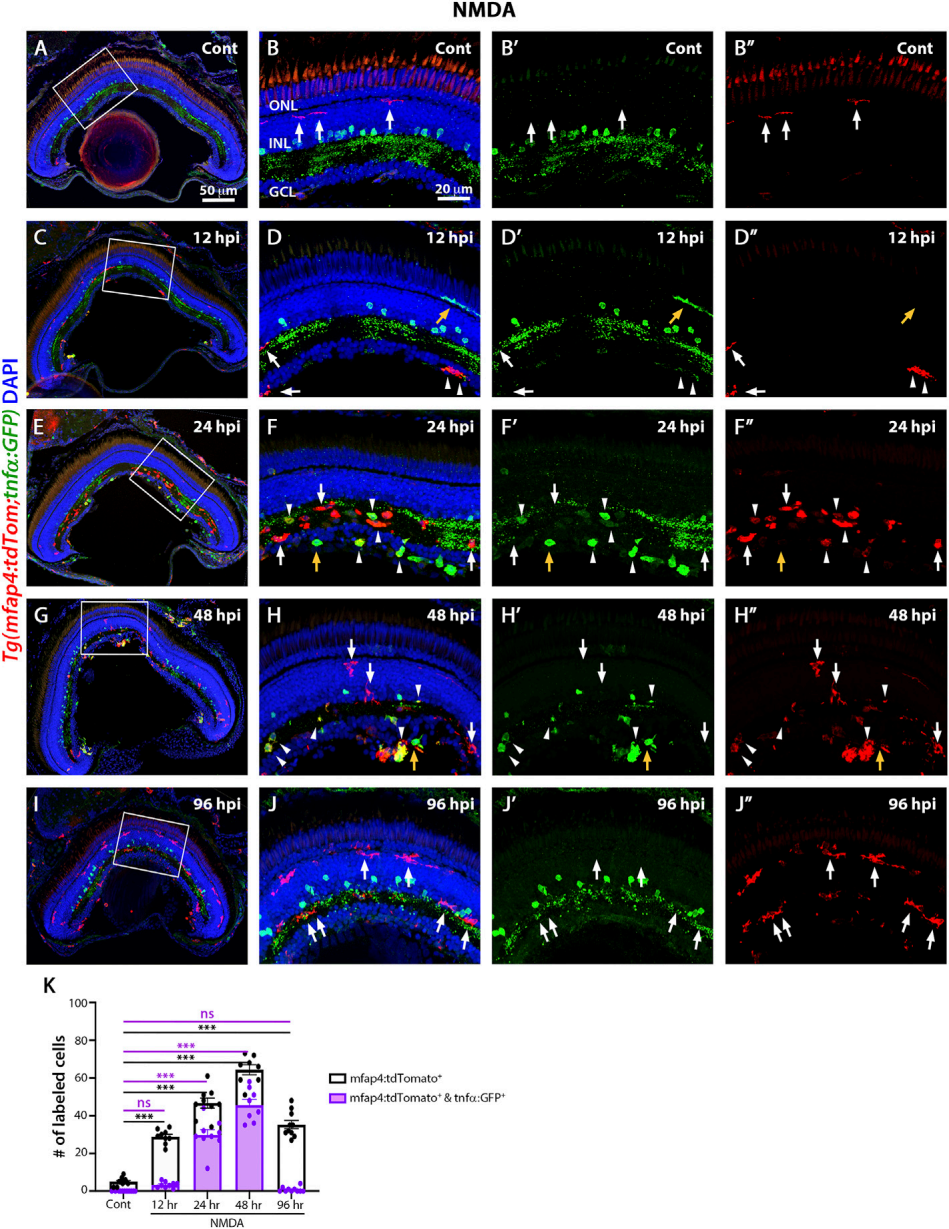
M1-like pro-inflammatory microglia are transiently identified in the NMDA-damaged acute injury model. Double-transgenic fish *Tg(mfap4:tdTomato;tnfα:GFP)* were used to monitor M1-like pro-inflammatory microglia/macrophages. Confocal images show 3 wpf control retinas possess tdTomato-positive ramified microglia (arrows), while amacrine cells express GFP **(A**,**B**–**B”)**. NMDA-injured retinas were evaluated at 12, 24, 48, and 96 h post-injection (hpi, **C**–**J**). At 12 h following NMDA injection, *mfap4:tdTomato*-positive microglia/macrophages start to co-express GFP (arrowheads). GFP-positive microglia/macrophages are detectable up to 72 hpi, but they are no longer detectable by 96 hpi. White boxes in Panels A, C, E, G, I show the area magnified in Panels B, D, F, H, J. White arrows: tdTomato-positive, GFP-negative microglia/macrophages; yellow arrows: tdTomato-negative, GFP-positive microglia/macrophages; arrowheads: tdTomato and GFP double-positive microglia/macrophages. Histogram displays the number of microglia/macrophages in 300 μm of the central region of the NMDA-damaged retinas **(K)**. Bars and lines indicate mean ± SEM, *n* = 8–12 per group. Black bars: total number of tdTomato-positive cells; purple bars: tdTomato and GFP double-positive microglia/macrophages. Statistical analysis was performed with a One-way ANOVA with Tukey’s multiple comparisons test was applied (*p < 0.05; **p < 0.001; ***p < 0.001).

The *TgBAC(tnfα:GFP)* reporter for TNFα expression, which is a pro-inflammatory cytokine and a well-established marker of pro-inflammatory macrophages, is informative in discriminating macrophage subsets in zebrafish (Nguyen-Chi et al., 2015). To identify pro-inflammatory macrophages, the *TgBAC(tnfα:GFP); Tg(mfap4:tdTomato-CAAX)* double-transgenic reporter line was intravitreally injected with NMDA (Figures 5A–J″) and evaluated at 12, 24, 48, and 96 hpi. Control retinas displayed tdTomato-positive, GFP-negative microglia/macrophages (Figures 5A–B″, K; 5 ± 0.56), which possessed thin and ramified processes. Additionally, based on cell localization and shape, GFP labeled a subset of amacrine cells, staining the cell body and neuronal projections with punctuate staining in the IPL (sublamina *a* and *b*) (Torvund et al., 2017). At 12 h after NMDA injection, there was a significant increase in the number of tdTomato-positive microglia/macrophages in the inner retina and they continued increasing in number and showed strong reactivity based on morphology by 24 hpi (Figures 5C–F″,K; 12 hpi: 28.89 ± 1.25 tdTomato-positive cells, *p* < 0.001, 24 hpi: 46.67 ± 2.67, *p* < 0.001). By 24 hpi, there was a significant increase in the number of tdTomato-positive microglia/macrophages coexpressing GFP compared to controls (Figures 5F–F″,K, arrowheads; 29.78 ± 2.83 tdTomato- and GFP- double positive cells, *p* < 0.05), with a majority of the tdTomato-positive microglia/macrophages expressing either high, medium or low levels of GFP. Surprisingly, a few GFP-positive and tdTomato-negative cells are also present, which could represent either a subset of microglia/macrophages that lost tdTomato expression or other immune cells, i.e., recruited neutrophiles. At 48 hpi, there was a significant increase in the number of GFP-positive pro-inflammatory microglia/macrophages relative to controls (Figures 5G–H″,K; 64.37 ± 2.65 tdTomato-positive cells; 45.63 ± 3.06 tdTomato- and GFP-double positive cells, *p* < 0.01). By 96 hpi, only a few tdTomato-expressing microglia/macrophages expressed GFP (Figures 5I–J″,K; NMDA 96 hpi: 35.3 ± 2.18 tdTomato-positive cells; 0.8 ± 0.42 tdTomato- and GFP-double positive cells), however, these microglia/macrophages had become more ramified rather than ameboid.

**FIGURE 6:**
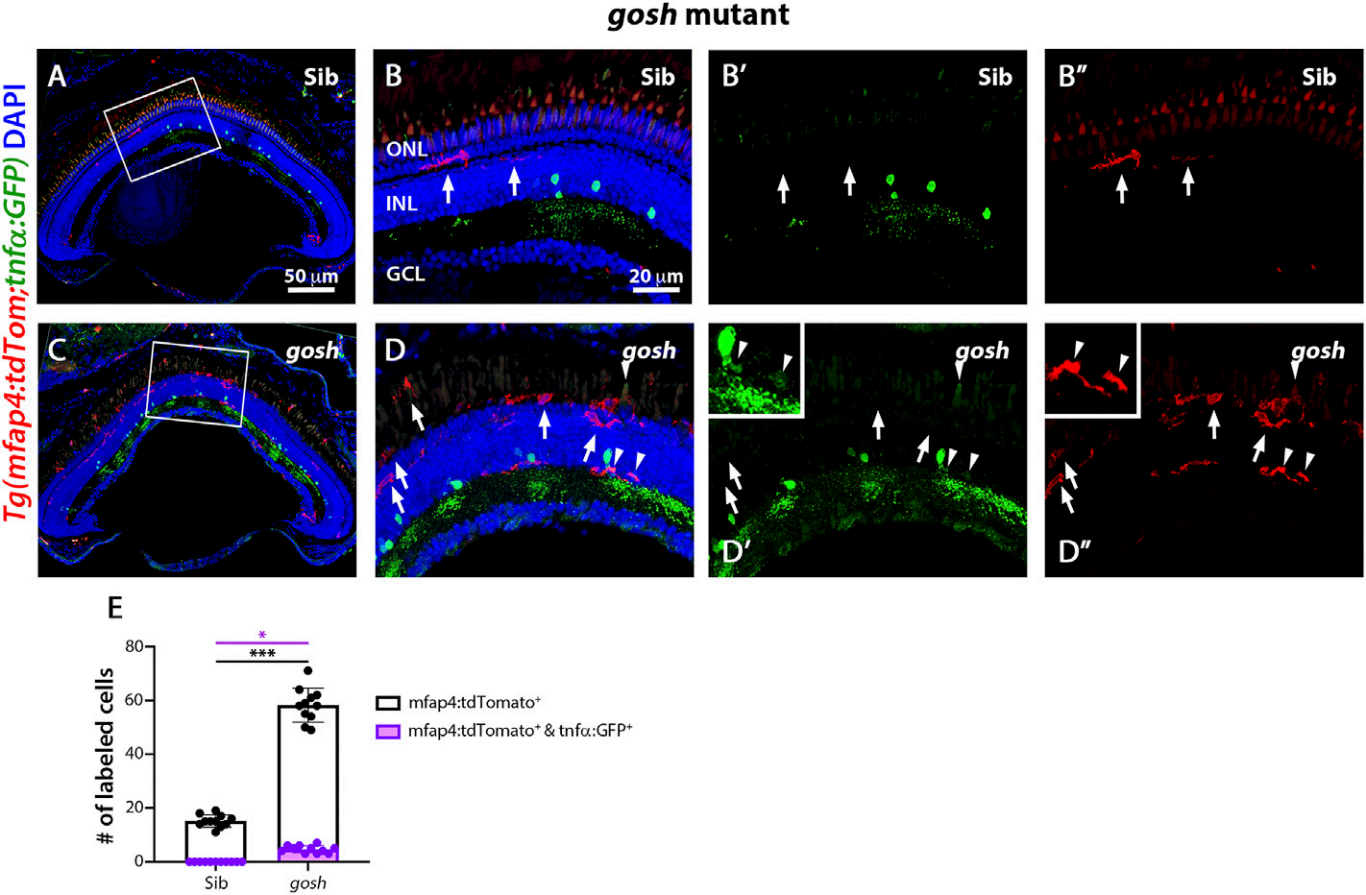
The *gosh* mutant chronic injury model possesses a small population of M1-like pro-inflammatory microglia/macrophages. At 3 wpf, control *gosh* sibling retinas display few and ramified tdTomato-expressing microglia/macrophages (**A**–**B”**, arrows). In contrast, *gosh* mutant retinas exhibit many ameboid microglia/macrophages in the ONL and outer segments layers, with only a few of these microglia/macrophages co-expressing GFP (**C**–**D”**, arrowheads). White boxes in Panels A and C show the area magnified in Panels **(B**,**D)**. White arrows: tdTomato-positive, GFP-negative microglia/macrophages; arrowheads: tdTomato and GFP double-positive microglia/macrophages. Histogram displays the number of microglia/macrophages in the entire section of the retinas **(E)**. Bars and lines indicate mean ± SEM, *n* = 11 per group. Black bars: total number of tdTomato-positive cells; purple bars: tdTomato and GFP double-positive microglia/macrophages. One-way ANOVA with Tukey’s multiple comparisons test was used for statistical analysis (*p < 0.05; ***p < 0.001).

The *gosh; TgBAC(tnfα:GFP); Tg(mfap4:tdTomato-CAAX)* mutants possessed a significantly greater number of amoeboid-shaped microglia/macrophages in the degenerating photoreceptor layer and outer segments compared to control *gosh* sibling retinas (Figure 6; sibling retinas: 15.18 ± 0.69 tdTomato-positive cells; *gosh* mutant: 58.27 ± 1.91, *p* < 0.001).

While the *gosh* mutant possessed a significantly greater number of tdTomato-positive microglia/macrophages co-labeled with GFP compared to the sibling controls (Figures 6C–D″,E, arrowheads; sibling retinas: 0 ± 0 tdTomato- and GFP-double positive cells; *gosh* mutant 4.64 ± 0.41, *p* < 0.05), it was a relatively small percentage of the tdTomato-positive microglia/macrophages. Thus, NMDA-injured retinas displayed a population of pro-inflammatory microglia/macrophages from 12 to 48 hpi that were no longer detected at 96 hpi, which might represent the switch from pro-inflammatory to anti-inflammatory/resolution of microglia/macrophages. On the other hand, *gosh* mutant microglia/macrophages showed an activated state, with a relatively small percentage expressing the pro-inflammatory cytokine TNFα.

### Modulating inflammation modifies microglia populations in acute and chronic retinal models

**FIGURE 7:**
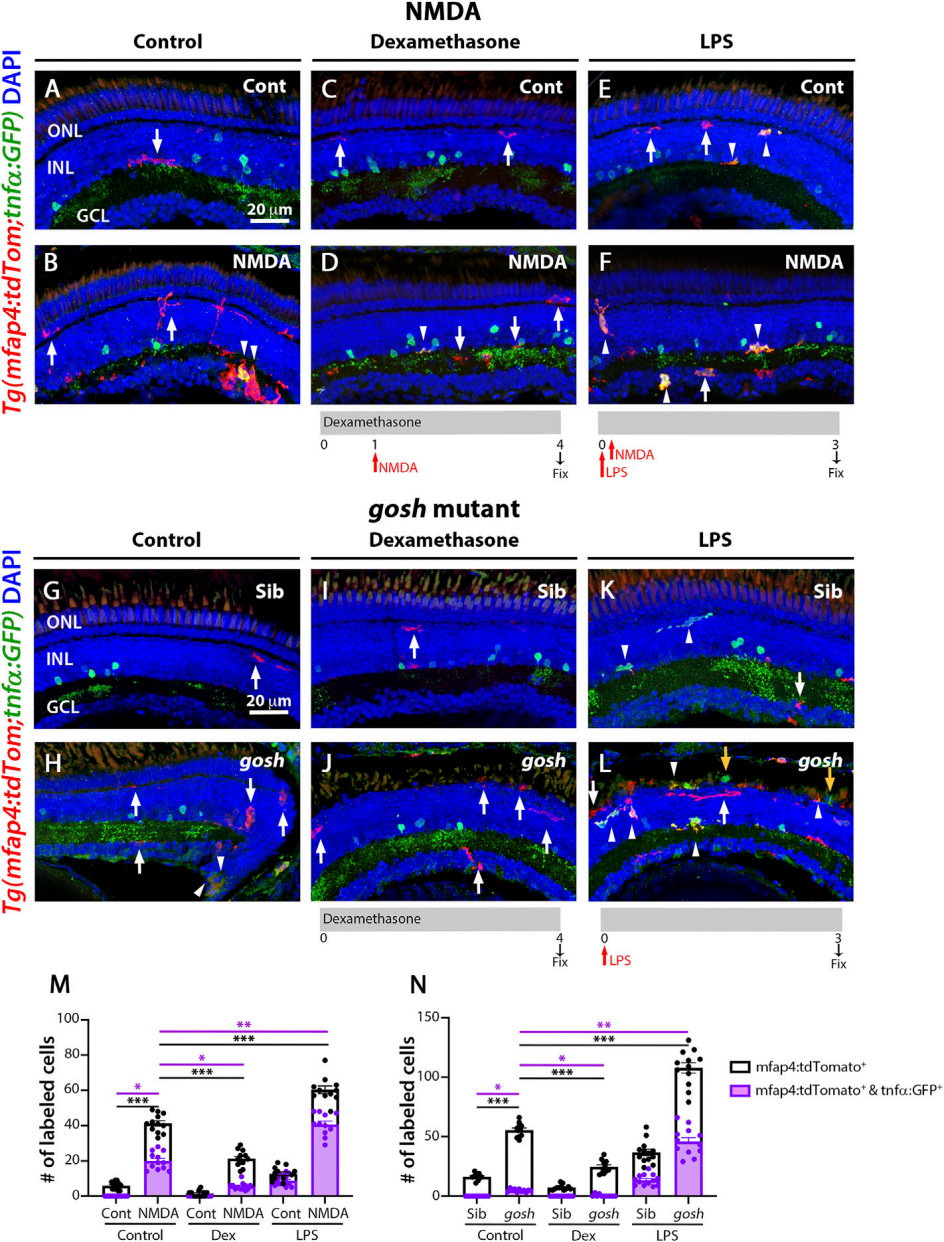
M1-like microglia are modulated by dexamethasone and LPS treatment in acute and chronic damaged retinas. At 3 wpf, *Tg(mfap4:tdTomato;tnfα:GFP)* fish, which allow visualization of M1-like pro-inflammatory retinal microglia/macrophages, were intravitreally injected with buffer **(A**,**C**,**E)** or NMDA **(B**,**D**,**F)**. A group of fish were placed in Dexamethasone, intravitreally injected with either buffer or NMDA 24 h later, and collected 72 h following the injections for immunostaining **(C**,**D)**. Alternatively, the fish were intravitreally injected with LPS, intravitreally injected 3 h later with either buffer or NMDA and collected 69 h later for immunostaining **(E**,**F)**. Control retinas possess some ramified tdTomato-positive microglia/macrophages (**A**, arrow). NMDA-injured retinas show significantly greater number of tdTomato-positive microglia/macrophages and tdTomato-positive cells that co-express GFP from the *tnfα:GFP* transgene (**B**, arrowhead; **M**). Dexamethasone treatment significantly reduces the number of tdTomato-positive microglia/macrophages and tdTomato-positive cells that co-express GFP (**D**, arrowhead; **M**). LPS treatment significantly increases the number of tdTomato-positive microglia/macrophages and tdTomato cells that colabel with GFP in control and NMDA-damaged retinas (**E**,**F**, arrowheads; **M**). *gosh* mutants display ameboid tdTomato-positive microglia/macrophages, and some also co-express GFP (**H**, arrowheads; **N**). Dexamethasone treatment significantly reduces the number of tdTomato-positive microglia/macrophages and tdTomato-positive cells that co-express GFP in control siblings **(I**,**N)** and *gosh* mutants **(J**,**N)**. LPS injection significantly increases the number of tdTomato-positive microglia/macrophages and GFP/tdTomato-double-positive cells in controls (**K**, arrows; **N**) and *gosh* mutants (**L**, arrowheads; **N**). White arrows: tdTomato-positive, GFP-negative microglia/macrophages; yellow arrows: tdTomato-negative, GFP-positive microglia/macrophages; arrowhead: tdTomato, GFP double-positive microglia/macrophages. Histograms represent the number of tdTomato-positive microglia/macrophages in 300 μm of the central region of the NMDA-damaged retinas **(M)** or the entire section of the *gosh* retinas **(N)**. Bars and lines indicate mean ± SEM, *n* = 10–13 per group. Black bars: total number of tdTomato-positive cells; purple bars: tdTomato and GFP double-positive microglia/macrophages. Statistical analysis was performed with One-way ANOVA with Tukey’s multiple comparisons test on both graphs (*p < 0.05; **p < 0.01, ***p < 0.001).

NMDA-injured and *gosh* mutant retinas treated with the anti-inflammatory drug dexamethasone displayed a low number of proliferating Müller glia (Figure 2), while the pro-inflammatory compound LPS increased the number of proliferating Müller glia (Figure 3). To assess whether dexamethasone or LPS affected the pro-inflammatory microglia/macrophages phenotype in injured retinas, we used the double transgenic *Tg(mfap4:tdTomato-CAAX)*; *TgBAC(tnfα:GFP)* reporter line. Undamaged control retinas possessed a low number of tdTomato-positive, GFP-negative microglia/macrophages (Figure 7A, M; control: 5.92 ± 0.57). In contrast, NMDA-induced retinal damage significantly increased the number of pro-inflammatory activated microglia/macrophages compared to the control (Figures 7B,M, arrowheads; 41.25 ± 1.51 tdTomato-positive cells, *p* < 0.001; 19.92 ± 1.44 tdTomato- and GFP-double positive cells, *p* < 0.05). Dexamethasone treatment significantly reduced the number of tdTomato-positive microglia/macrophages that did not co-label with GFP and those that co-labeled with GFP (Figures 7C,D,M; 21.25 ± 1.33 tdTomato-positive cells, *p* < 0.001; 5.5 ± 0.61 tdTomato- and GFP-double positive cells, *p* < 0.05). In contrast, LPS treatment significantly increased the number of tdTomato-positive microglia/macrophages and the number of tdTomato- and GFP-double positive pro-inflammatory microglia/macrophages (Figures 7E,F,M, arrowheads;tdTomato-positive cells: 60.36 ± 2.17 *p* < 0.001; tdTomato- and GFP-double positive cells: 40.64 ± 1.91, *p* < 0.01).

Sibling and *gosh* mutant retinas possessed tdTomato-positive microglia/macrophages, while only *gosh* mutant displayed tdTomato- and GFP- double positive cells (Figures 7G,H,N; sibling retinas: 16.3 ± 0.91 tdTomato-positive cells; 0 ± 0 tdTomato- and GFP-double positive cells; *gosh* mutant: 55.55 ± 1.77; 4.82 ± 0.38 tdTomato- and GFP-double positive cells). Upon dexamethasone treatment, *gosh* retinas possessed significantly fewer tdTomato-positive microglia/macrophages and tdTomato- and GFP-double positive cells than controls (Figures 7I,J,N; 24.63 ± 1.68 tdTomato-positive cells, *p* < 0.05; 0.46 ± 0.31 tdTomato- and GFP-double positive cells, *p* < 0.001). LPS treatment significantly increased the number of tdTomato-positive microglia/macrophages and the number of tdTomato- and GFP-double positive pro-inflammatory microglia/macrophages in the *gosh* mutant compared to controls (Figures 7K,L,N, arrowheads; 107.8 ± 4.38 tdTomato-positive cells, *p* < 0.01; tdTomato- and GFP-double positive cells, 45.92 ± 3.37, *p* < 0.001). Taken together, these data suggested that modulating the inflammatory response *via* either dexamethasone or LPS treatment not only affected the total number of microglia/macrophages in both acute and chronic retinal damage models, but also the number of M1-like pro-inflammatory microglia/macrophage cells.

## Discussion

**FIGURE 8:**
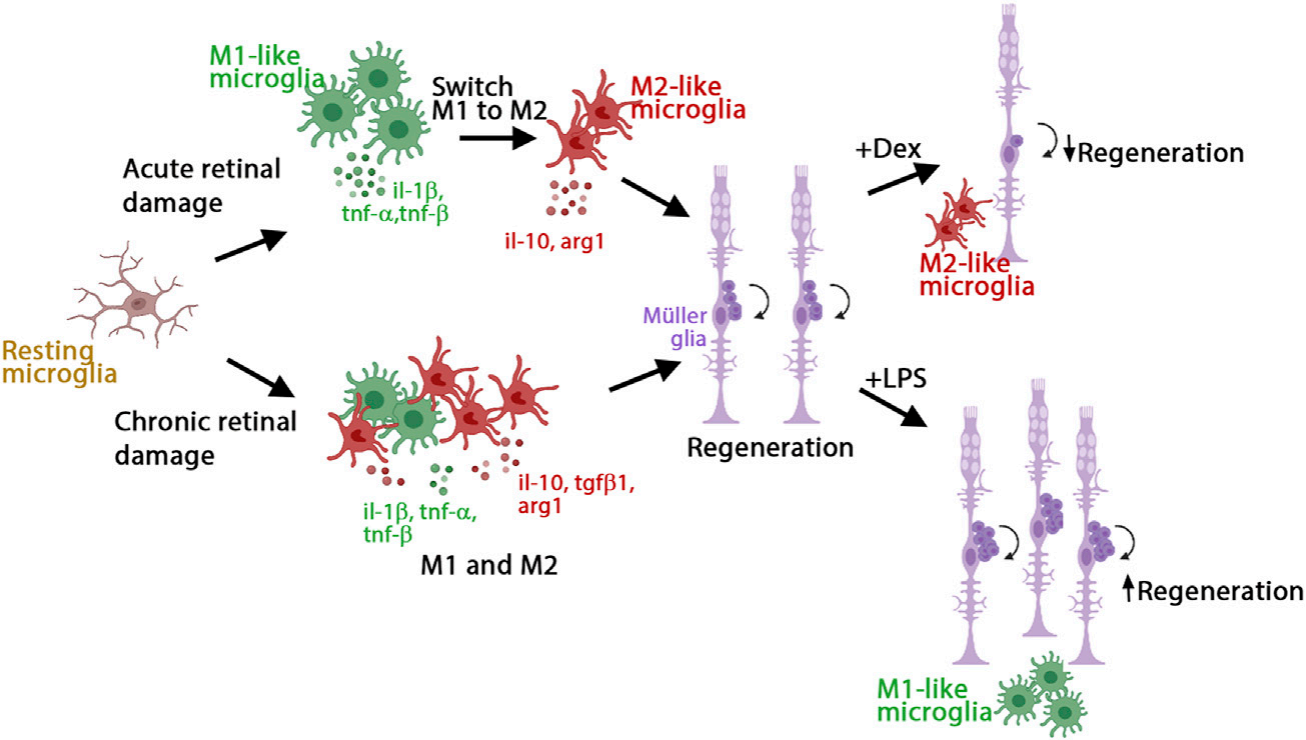
Model of inflammation regulating the regenerative response in acute and chronic retinal damage in zebrafish. Diagram representing microglia/macrophages activation and polarization upon NMDA-acute injury or *gosh* chronic mutant retinal damage in zebrafish to induce regeneration. This acute injury model stimulates an early pro-inflammatory response, which then switches to an anti-inflammatory profile. This chronic injury model stimulates a dominant anti-inflammatory response, accompanied by a mild pro-inflammatory response. Modulating inflammation can inhibit or amplify the regeneration capacity *via* regulating the presence of M1-like pro-inflammatory microglia/macrophages.

We investigated the role of inflammation in the initiation of retina regeneration following either an acute or chronic injury model in 3 wpf zebrafish. We showed that these different injury paradigms induced inflammation and proliferation of Müller glia and NPCs and modulating inflammation regulated the proliferative response. NMDA-injured acute retinal damage induced an early upregulation of pro-inflammatory cytokines, followed by increased expression of anti-inflammatory cytokines. In addition, TNFα-expressing M1-like pro-inflammatory microglia/macrophages were transiently present during the regenerative response. In contrast, the *gosh* chronic retinal damage mutant upregulated a robust anti-inflammatory response in the presence of a mild pro-inflammatory response. TNFα-expressing M1-like microglia/macrophages were modulated by dexamethasone and LPS treatment in both of the acute and chronic injury models used in this study, which affected Müller glia proliferation (Figure 8). While both acute and chronic retinal damage induced a regeneration response (Figure 8), the magnitude of the regeneration response was larger in the acute damage model, possibly due to a greater number of dying retinal neurons. It would be interesting to evaluate different damage paradigms and determine if this study’s findings were consistent with other acute and chronic retinal damage models. It is possible that a different immune response arises based on the total number of neurons or different types of neurons lost.

Tissue injury elicits a rapid and strong immune response that is required for regeneration in zebrafish (Marz et al., 2010; Baumgart et al., 2012; Lai et al., 2019; Iribarne, 2021; Nagashima and Hitchcock, 2021). Recent research showed that knockdown of macrophage differentiation impaired fin regeneration in larvae and genetic ablation of macrophages affected fin growth in adult zebrafish (Li et al., 2012; Petrie et al., 2014). Blocking inflammation in spinal-lesioned zebrafish larvae reduced axonal bridging and GFAP-positive proliferating radial glia (Ohnmacht et al., 2016; Tsarouchas et al., 2018), whereas activation of the immune system increased axonal regeneration (Tsarouchas et al., 2018). Inducing acute inflammation followed by an optic nerve crush injury increased Müller glia proliferation and accelerated axonal re-growth, while depleting retinal microglia and macrophages impaired optic nerve regeneration (Van Dyck et al., 2021). Depleting microglia or inhibiting their functions in damaged larval and adult zebrafish retinas blocked Müller glia regenerative responses (White et al., 2017; Silva et al., 2020; Zhang et al., 2020). Our results are consistent with these results, as the injury paradigms used in this research induced damage that triggered microglia/macrophage reactivity. Moreover, inhibiting the inflammatory response or ablating macrophages reduced the number of proliferating Müller glia and NPCs, while activating the inflammatory response increased the number of proliferating Müller glia and NPCs in both acute and chronic retinal injury models. These results suggest that macrophages play a critical role in zebrafish tissue regeneration.

Macrophages exist in a variety of acquired states under different environments. In our acute damage model, TNFα-expressing M1-like pro-inflammatory macrophages transiently accumulated early in regeneration. As expected, the expression of pro-inflammatory cytokine genes was upregulated early, followed by the upregulation of anti-inflammatory cytokines. In zebrafish, a limited number of studies described the presence of different macrophage subtypes during the regeneration process. Petrie and colleagues showed that macrophages at different stages are responsible for different functions during fin regeneration (Petrie et al., 2014). Early and transient recruitment of TNFα-expressing pro-inflammatory macrophages occurred during fin regeneration (Nguyen-Chi et al., 2017). More recent studies identified a transient pro-regenerative macrophage subtype in zebrafish with a specific gene expression profile (Sanz-Morejon et al., 2019; Cavone et al., 2021; Ratnayake et al., 2021). In the regeneration of the larval spinal cord, the pro-regenerative macrophage population expressed TNFα (Cavone et al., 2021), however the pro-regenerative macrophage population was TNFα-negative and Wilms-positive in heart and fin regeneration models (Sanz-Morejon et al., 2019). In contrast, a cytokine nicotinamide phosphoribosyltransferase (NAMP)-positive pro-regenerative macrophage population was identified during muscle regeneration (Ratnayake et al., 2021). At least the TNFα and Wilms pro-regenerative macrophage subtypes were different populations, suggesting that specific markers and macrophage subtypes are associated with regenerating different tissues. These results indicate that an inflammatory response followed by resolution is required to achieve successful regeneration, involving different macrophage subtypes in each stage. It has yet to be elucidated if there is only one macrophage subtype culpable for the scope of functions during inflammation and if there is a switch from pro-inflammatory to an anti-inflammatory profile, as was observed *in vivo* in zebrafish larvae (Nguyen-Chi et al., 2015), or whether several macrophage subtypes are involved.

Mammals undergoing chronic degeneration suffer not only injury-induced primary damage, but also prolonged inflammatory activation inducing secondary damage, which could be more detrimental than the primary injury itself (Noailles et al., 2016; Zabel et al., 2016). It has been hypothesized that inflammatory macrophages or microglia damage the CNS, while a resolving phenotype contributes to neuroregeneration (Kigerl et al., 2009). Upon traumatic spinal cord injury in rats, the M1-like pro-inflammatory macrophage response was rapidly induced and maintained at damage sites. This response overwhelmed a comparatively smaller and transient M2 macrophage response. Interestingly, *gosh* mutant zebrafish suffering chronic photoreceptor degeneration exhibited inflammation that had at least two components; a mild pro-inflammatory component and a stronger anti-inflammatory component. The specific cytokine environment could explain the surprising success in regeneration during chronic damage in zebrafish. The ratio between M1- and M2-like macrophages could have significant implications for CNS repair. Specifically, in zebrafish, the necessity of M1-like pro-inflammatory microglia is apparent by the inhibition of this population preventing Müller glia proliferation (Figure 7). Alternatively, it could be that the transient pro-regenerative TNFα expressing microglia subtype is sufficient to induce regeneration, similar to the TNFα pro-regenerative microglia subtype stimulating spinal cord regeneration (Cavone et al., 2021). To our knowledge, our study is the first that focused on the role of microglia in modulating the regenerative response of a chronic injury in zebrafish.

A transient inflammatory response mediated by IL-1β is required for proper regeneration in the zebrafish fin fold (Hasegawa et al., 2017). Macrophages attenuated IL-1β expression and inflammation to support the survival of regenerative cells. Furthermore, inflammation mediated by IL-1β was necessary for normal fin regeneration by triggering the expression of regeneration-induced genes (Hasegawa et al., 2017). In the resected spinal cord, early expression of IL-1β promoted axonal regeneration, while prolonged high levels of IL-1β were detrimental. Another study showed that TNFα was one of the critical signals transiently expressed by polarized macrophages during the early phases of fin regeneration (Nguyen-Chi et al., 2017). The proliferation of stromal cells depended on TNFα/TNFr1 signaling, suggesting that IL-1β and TNFα are expressed at different times, with IL-1β being expressed earlier and followed by pro-inflammatory macrophages expressing TNFα (Nguyen-Chi et al., 2017). We found that NMDA-induced acute retinal injury stimulated an early peak of IL-1b expression, followed by increased expression of TNFa and TNFb during retinal regeneration (Figure 4B). Although it remains unclear if a similar mechanism is present in both the fin and spinal cord tissues, these results suggest that the activation and duration of pro-inflammatory signals and the subsequent resolution are critical in creating an instructive microenvironment for tissue regeneration.

In conclusion, we found that the NMDA-induced acute and *gosh* mutant chronic damage models stimulated a regenerative response *via* inducing different pro-inflammatory response strategies. NMDA-acute damage induced an early and transient pro-inflammatory response, whereas *gosh* mutant chronic damage stimulated a persistent mild pro-inflammatory response in the presence of a stronger anti-inflammatory response. Additionally, pro-inflammatory microglia/macrophages are required for the regenerative response, as their abolition impaired regeneration. Understanding how inflammation regulates regeneration in the injured zebrafish retina would provide important insights to improve the therapeutic strategies for repairing injured mammalian tissues lacking an inherent regenerative capacity.

## Data Availability

The original contributions presented in the study are included in the article/Supplementary Material, further inquiries can be directed to the corresponding author.

## Supplementary material

Supplementary Figure S1 NMDA damage recruits microglia and a few peripheral macrophages. Seventy-two hours after injecting either water (control) or NMDA intravitreally into transgenic *Tg(mpeg1:eGFP)* retinas, retinas were stained for eGFP, which labels microglia/macrophages, and with the 4C4 antibody to label microglia, but not peripheral macrophages. Nuclei were labeled with DAPI (blue). In control retinas, eGFP and 4C4 label the same population of cells (**A–C**, white arrows). NMDA-injured retinas display most eGFP-positive cells co-label with 4C4 (**D–F**, white arrows). Few cells are infiltrated peripheral macrophages (yellow arrows, inset). White arrow: 4C4- and eGFP-double-positive cells; yellow arrow: eGFP-positive cells (one cell shown in the inset); arrowhead: 4C4-positive cells; asterisks in the inset: eGFP22 positive cells.

Supplementary Figure S2 Microglia and a few peripheral 1 macrophages are detected in the *gosh* mutant. Three-wpf wild-type sibling and *gosh* mutant retinas combined with the transgene *Tg(mpeg1:eGFP)* were stained with the 4C4 antibody and nuclei were labeled with DAPI (blue). In wild-type retinas, eGFP and 4C4 colabeled microglia (A–C, white arrow). *gosh* mutants possessed eGFP and 4C4 colabeled microglia (D–F, white arrows), as well as a few eGFP-positive, 4C4-negative cells, which represented peripheral macrophages (yellow arrows, inset). White arrow: 4C4- and eGFP-double-positive cells; yellow arrow: eGFP-positive cells (one cell shown in the inset); arrowhead: 4C4-positive cells; asterisks in the inset: eGFP10 positive cells.

Supplementary Figure S3
*gosh* mutant exhibits signs of inflammation at 1, 2, and 4 wpf. Confocal images of control and *gosh* mutants combined with the transgene *Tg(mpeg1:eGFP)* at 1, 2, and 4 wpf **(A–F)**. Nuclei were stained with DAPI (blue). Control retinas displayed few, thin, and ramified microglia/macrophages **(A–C)**. *gosh* mutant retinas exhibited a greater number of ameboid-shaped/activated microglia/macrophages, located primarily in the outer retina at all the time points evaluated **(D–F)**. Head samples of control or *gosh* mutant fish at 1, 2, and 4 wpf were used to evaluate the fold-change of mRNA expression by quantitative realtime PCR of *mpeg1* and *p2ry12* **(G–I)**; *il-1 b*, *tnf a*, and *tnfb* **(J–L)**; *il-10*, *tgf-b1*, *ccr2*, and *arg1* **(M–O)**. All the genes evaluated show a similar pattern of expression compared to the different time points, except for *tgf-b1* at 1 wpf.
