# Supplementary material for: Different inflammation responses modulate Müller glia proliferation in the acute or chronically damaged zebrafish retina
Source: Front Cell Dev Biol. 2022 Aug 31;10:892271. doi: 10.3389/fcell.2022.892271 (PMC9472244; doi:10.3389/fcell.2022.892271)
Supplement: Supplementary file 1 [file DataSheet1.pdf]

Supplementary Materials

Different inflammation responses modulate Müller glia proliferation in the acute or chronically damaged zebrafish retina

Maria Iribarne and David R. Hyde

Supplementary Figures: Figures S1, S2, S3

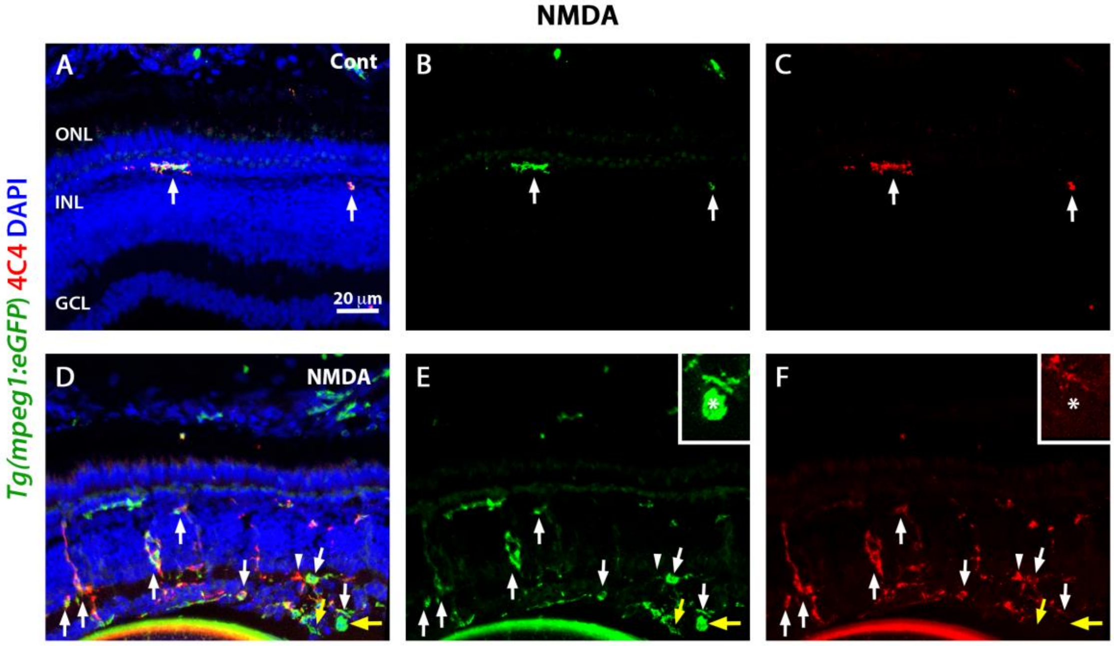

Supplementary Figure 1. NMDA damage recruits microglia and a few peripheral macrophages.

Seventy-two hours after injection, control and NMDA-injured retinas expressing the transgene *Tg(mpeg1:eGFP*), which visualizes microglia/macrophages, were stained with the antibody against 4C4 to label microglia, but not peripheral macrophages. Nuclei were labeled with DAPI (blue). In control retinas, eGFP and 4C4 label the same population of cells (A-C, white arrows). NMDA-injured retinas display most GFP-positive cells that are co-label with 4C4 (D-F, white arrows). Few cells are infiltrated peripheral macrophages (yellow arrows, inset). White arrow: 4C4- and eGFP-double-positive cells; yellow arrow: eGFP-positive cells (one cell shown in the inset); arrowhead: 4C4-positive cells; asterisks in the inset: eGFP-positive cells.

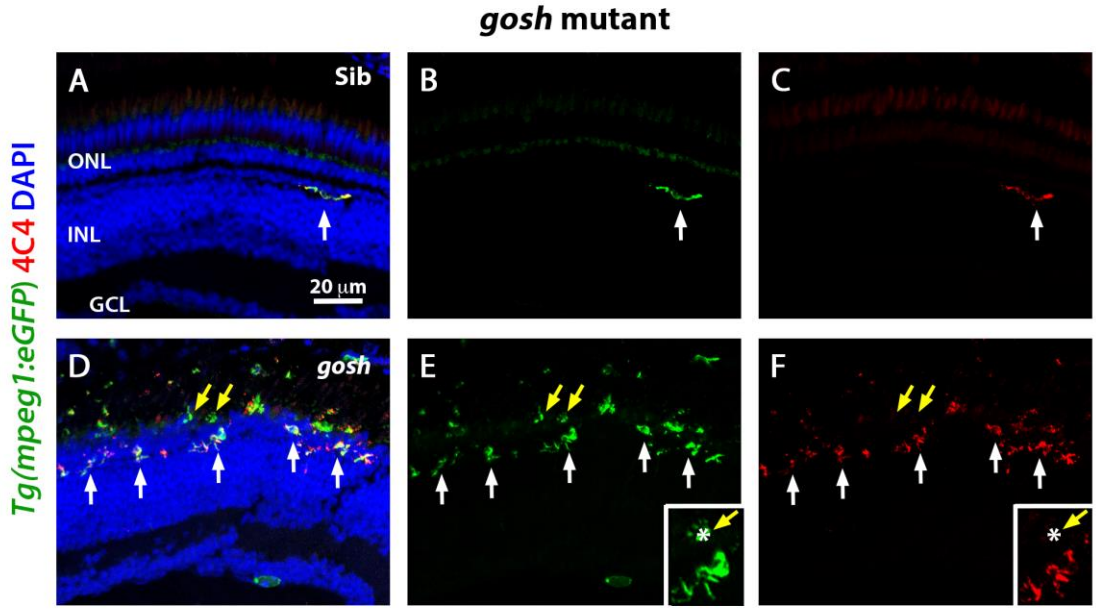

Supplementary Figure 2. Microglia and a few peripheral macrophages are detected in the *gosh* mutant.

Three-wpf wild-type sibling and *gosh* mutant retinas combined with the transgene *Tg(mpeg1:eGFP*) were stained with the antibody against 4C4. Nuclei were marked with DAPI (blue). In wild-type retinas, eGFP and 4C4 labels are observed in microglia (A-C, white arrow). *gosh* mutants show GFP-positive cells stained with 4C4 (D-F, white arrows). Few cells are stained only with eGFP, which indicates that they are peripheral macrophages (yellow arrows, inset). White arrow: 4C4- and eGFP-double-positive cells; yellow arrow: eGFP-positive cells (one cell shown in the inset); arrowhead: 4C4-positive cells; asterisks in the inset: eGFP-positive cells.

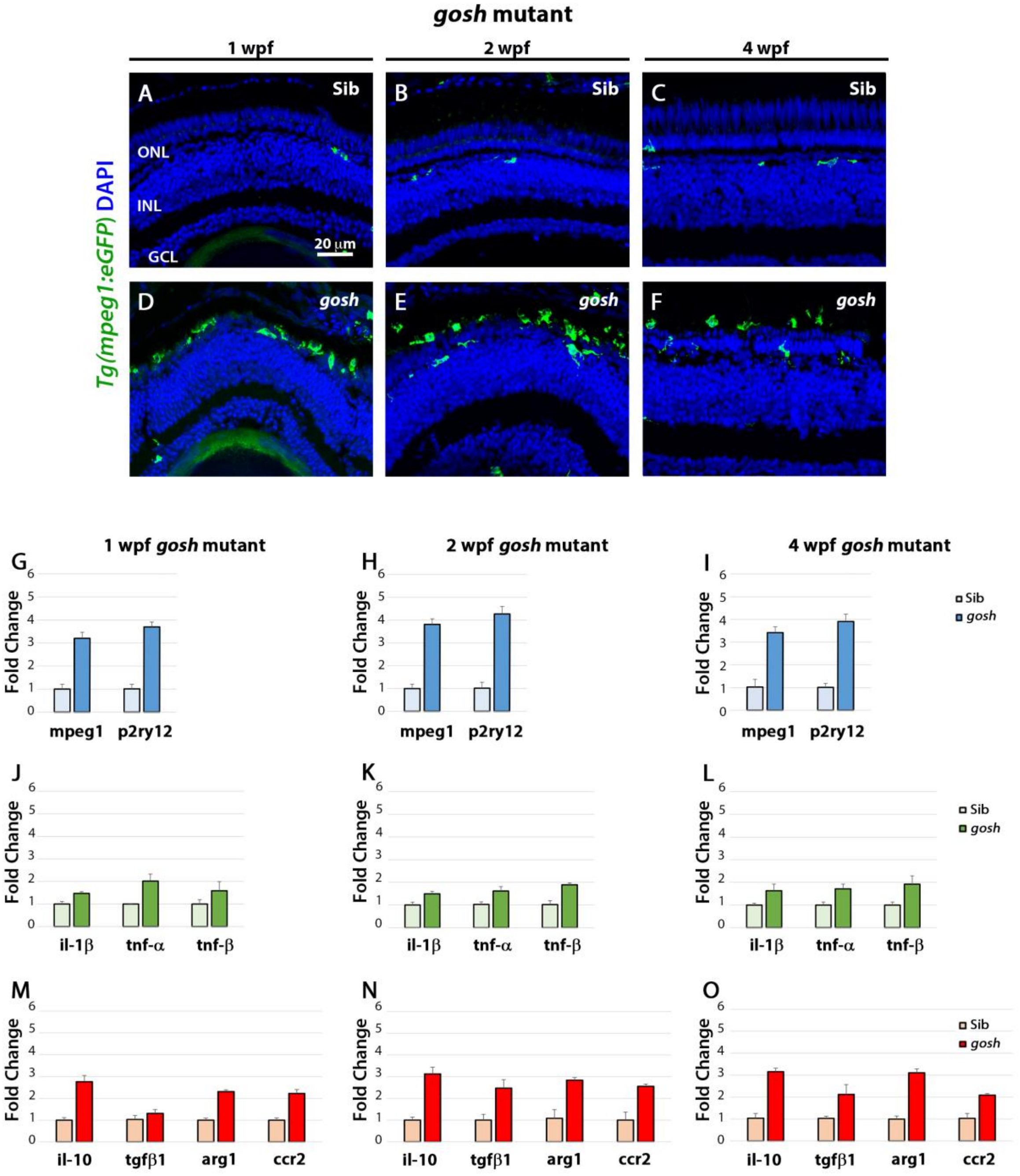

Supplementary Figure 3. ***gosh*** **mutant exhibits signs of inflammation at 1, 2, and 4 wpf.**

Confocal images of control and *gosh* mutant combined with the transgene *Tg(mpeg1:eGFP*) at 1, 2, and 4 wpf (A-F). Nuclei were stained with DAPI (blue). Control retinas display few, thin, and ramified microglia/macrophages (A-C). *gosh* mutant retinas exhibit a high number of microglia/macrophages with ameboid-shaped, located mainly in the outer retina at all the time points evaluated (D-F). Head samples of control or *gosh* mutant samples at 1, 2, and 4 wpf

were used to evaluate the fold-change of mRNA expression by quantitative real-time PCR of *mpeg1* and *p2ry12* (G-I); *il-1β, tnfα,* and *tnfβ* (J-L); *il-10, tgf-β1*, *ccr2*, and *arg1* (M-O). All the genes evaluated show a similar pattern of expression compared to the different time points, except for *tgf-β1* at 1 wpf.
